# Calcium Carbonate Urolithiasis in a Pediatric Patient: A Case Report

**DOI:** 10.7759/cureus.47873

**Published:** 2023-10-28

**Authors:** Jose L Millet-Herrera, Ricardo Méndez-Molina, Andrea M Milke-Garcia, Teresa N Cruz-May, Nina Mendez-Dominguez, Juan P Flores-Tapia

**Affiliations:** 1 Clinical Sciences, Universidad Marista de Merida, Merida, MEX; 2 Clinical sciences, Universidad Marista de Merida, Merida, MEX; 3 Applied Physics, Centro de Investigación y de Estudios Avanzados del Instituto Politécnico Nacional, Merida, MEX; 4 Research, Hospital Regional de Alta Especialidad de la Peninsula de Yucatan, Merida, MEX; 5 Nephrology, Urology and Renal Transplant, Hospital Regional de Alta Especialidad de la Peninsula de Yucatan, Merida, MEX

**Keywords:** staghorn renal stone, case report, pediatric urolithiasis, calcite, calcium carbonate, urolithiasis

## Abstract

Urinary stones composed of calcium carbonate are extremely rare, accounting for 0.01%-1.4% of urolithiasis (UL) cases. Urolithiasis is an infrequent condition in the pediatric population worldwide and in Mexico; nevertheless, the incidence in the Yucatán Peninsula is higher than that reported in other areas of Mexico and the world. Urolithiasis is the second most common urinary disease among pediatrics in the Yucatán Peninsula, which makes it an endemic region for this disease. We describe the case of a five-year-old male from the southeast region of Mexico who presented with signs and symptoms of urinary tract infection (UTI) and was diagnosed with bilateral staghorn stones of calcium carbonate, successfully treated by mini endoscopic combined intra-renal surgery, and dietary adjustments to prevent recurrence.

## Introduction

Urolithiasis (UL) is a common condition among adults, with a global prevalence of up to 20% within this demographic; conversely, its occurrence among pediatrics accounts for only 2%-3% of the total cases, rendering it rare [1-3]. In Mexico, pediatric UL is also an uncommon disease, and the percentage of pediatric patients who undergo surgical treatment is very small [4]. Nevertheless, in the Yucatán Peninsula (located in the southeastern region of Mexico), UL represents 25.6% of pediatric urinary tract diseases, which makes it the second most common urological pathology among this age group, and therefore, it is considered an endemic area for pediatric UL [5]. This endemicity has been attributed to the convergence of genetic, environmental, dietary, and climatological factors that promote renal stone formation [6]. In terms of stone composition, calcium carbonate (calcite) calculi are very infrequent, as they are present in just 0.01%-1.4% of all UL cases, and pure calcite composition is even rare [7,8]. Calculi composition is significant for therapeutic planning, prognosis determination, and long-term management [9], but the clinical presentation of calcite-dominant and pure calcite UL in the pediatric population is unexplored, making its documentation crucial to guide optimal therapeutic approaches. Therefore, this case report aims to describe the clinical presentation of bilateral renal lithiasis of calcium carbonate in a pediatric patient from the southeastern region of Mexico.

## Case presentation

A five-year-old male from the Yucatán Peninsula in Mexico presented with fever, malaise, and dysuria. There was a family history of UL on the patient’s father and paternal grandparents. The child's growth status was within normal/high limits according to his age, with a height of 113 centimeters, or 44.5 inches, which is between the 75 and 90 percentile; in terms of weight, the patient's BMI-for-age percentile was located between the 89 and 95 percentiles (17 kg/m²), classifying him as overweight. Dietary habits were characterized by a high-calorie intake, frequent consumption of hypersodic foods, and inadequate hydration.

**Figure 1:**
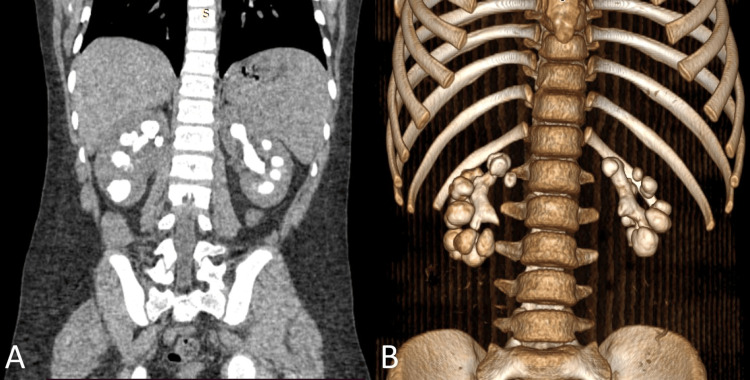
A) CT scan of the coronal section; B) 3D reconstruction of a CT scan, anterior view

Due to the presenting symptoms, an initial urinalysis was conducted, which revealed pyuria, hematuria, proteinuria, and a pH of 6.7 (alkaline). The patient's serum creatinine level was 0.61 mg/dL, but renal damage was detected because of the presence of proteinuria in the urinalysis. Because of the family history of UL and the endemicity of the disease in Yucatán, a suspicion of urolithiasis was raised, and therefore renal ultrasound was performed, where hyperechoic images with acoustic shadowing were observed in both kidneys, suggestive of UL. Given these findings, an abdominopelvic CT scan and 3D reconstruction were obtained (Figures 1A, 1B) to confirm the UL diagnosis, and its severity, and provide information for surgical planning.

Bilateral hyperdense renal images encompassing the entire collecting system were observed, confirming the diagnosis of bilateral staghorn calculi.

**Figure 2:**
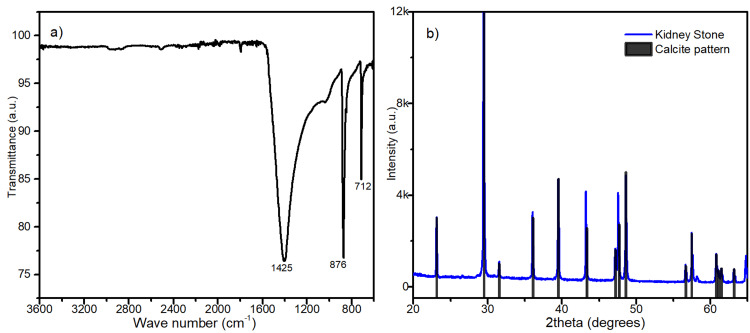
Chemical analysis results: A) Infrared spectroscopy; B) X-ray powder diffraction pattern

A staged surgical management with bilateral mini-endoscopic combined intra-renal surgery (ECIRS) was successfully performed, and the stone analysis conducted through spectroscopy (Figure 2A) and X-ray powder diffraction (Figure 2B) indicated the presence of calcite and a mineral phase of pure calcite, respectively.

Complementary, a 24-hour urine metabolic analysis was conducted, and hypercalciuria, hypomagnesuria, and hypocitraturia were identified (Table 1).

**Table 1:** Twenty-four-hour urine metabolic study Pt: patient; M, 5y: 5-year-old male; Ref: reference range; Vol: volume; Prot: proteins; Ca: calcium; Ox: oxalate; Cit: citrate; Na: sodium; Cl: chlorine; Mg: magnesium

Pt	Vol (ml)	pH	Prot (mg/24 h)	Ca (mg/24 h)	Ox (mmol/24 h)	Cit (mg/24 h)	Na (mEq/24 h)	Cl (mEq/24 h)	Mg (mg/24 h)
M, 5y	2,810	6.5	406.6	415.3	0.4	<56	134.9	130.3	65.0
Ref	-	5.5-6.5	0-140	100-300	0.14-0.42	100-1,300	40-220	170-245	70-122

These alterations were addressed by implementing an appropriate diet and administering oral potassium citrate, with good results after treatment.

## Discussion

A case of a five-year-old male from the Yucatán Peninsula diagnosed with bilateral staghorn lithiasis composed of pure calcite was presented. Globally, the incidence of pediatric UL constitutes a small fraction of total UL cases [5], and calcite calculi composition is extremely rare. Consequently, the occurrence of a calcite stone in a pediatric patient is quite exceptional.

Urolithiasis in the pediatric population is a growing disease, with studies showing up to a five-fold increase [10]. Despite that, urinary stones in pediatrics are still considered an uncommon disease worldwide, as they account for just 2%-3% of total UL cases [3]. However, in Yucatán, UL stands out as a very frequent cause of pediatric urinary consultations, and it is considered an endemic area for urinary stone disease, with pediatric UL accounting for 25.6% of the total pediatric urinary diseases [5,6]. In this region, its overall prevalence (including adults) is 5.5%-10.3%, which is 10 to 100 times higher than what is reported in the literature [11]. Unfortunately, there is a lack of research about it in the pediatric population of Yucatán, which makes it difficult to establish the prevalence of the disease in children.

In the Yucatán Peninsula, the presence of both modifiable and non-modifiable risk factors makes its inhabitants more susceptible to kidney stone formation [6]. It has been reported that the most important risk factors for the development of pediatric UL are metabolic disorders. In particular, in the population of the Yucatán Peninsula, a deficiency of UL inhibitors is very prevalent, among which hypocitraturia stands out as the most relevant, found in 91.3% of individuals, also associated with other disturbances like hypomagnesuria (68.5%), hypercalciuria (42.1%), hyperuricemia (33.3%), hyperuricosuria (26.6%), and hyperoxaluria (36.5%) [6]. Along with the mentioned metabolic disturbances, high temperatures, and humidity levels in the region may lead to chronic dehydration and subsequently urine supersaturation, which explains the higher prevalence of UL. It is important to mention that, considering his young age, genetics could also be involved in the development of UL in this pediatric patient; the family history of UL on the father’s side raises suspicion about a genetic component. However, there is no information on risk factors for UL specifically in the pediatric population in Yucatán, therefore, research needs to be done.

Regarding stone composition, according to the cohort study conducted by Degheili et al. in 2022, which analyzed the stone composition of 624 patients, it was found that calcium carbonate stones accounted for only nine (1.4%) cases, which makes it very rare [7]. The pathophysiology of urinary stones in general is multifactorial; unfortunately, because of their rarity, there is not so much information about the pathogenesis and risk factors related to calcite stones, and the literature is limited to reporting that this composition is often associated with urinary tract infections [10]. Moreover, renal stones composed of just one type of mineral (as reported in this manuscript) are uncommon, as demonstrated in a study carried out in the endemic region of the Yucatán Peninsula by Rodríguez-Plata et al. in 2021, where thirty samples of renal stones were analyzed and none of them turned out to have a pure composition; they all had two or three components; in that study, the predominant compound was calcium oxalate dihydrate [9], which is the most common type of stone in adults and pediatrics [12].

Over the past 25 years, in Mexico, pediatric lithiasis has exhibited a yearly upward trend of 6%-10%, particularly in areas where it is considered endemic. This underscores the significance of considering UL as a potential diagnosis in pediatric patients who frequently present with symptoms of urinary tract infection, abdominal pain radiating to the scrotum or labia, fever, microscopic or macroscopic hematuria, nausea, and vomiting [13]. The first step in the diagnosis of UL would be to identify the stone by imaging; renal ultrasound is a good first-choice diagnostic tool because of the absence of radiation for pediatric patients; nevertheless, depending on the location of the calculi, they are sometimes difficult or impossible to visualize [12]. On the other hand, non-contrast computed tomography remains the gold standard and is indicated in children without a diagnosis after ultrasound and a high suspicion of UL, as CT can confirm the UL diagnosis and be beneficial for surgical planning. Subsequently, it is necessary to perform an analysis of the calculus by infrared spectroscopy or X-ray diffraction analysis to identify the mineral composition.

Some authors consider pediatric UL as a symptom rather than a disease; therefore, when addressing the diagnosis of lithiasis in pediatric patients, it is essential to conduct an early and comprehensive approach that combines metabolic studies, nutritional interviews, and, in feasible cases, analysis of the mineral composition of the calculi to determine what is causing the child to develop stones. Urinary stones developed during childhood have a risk of recurrence of up to 20%, and those with metabolic disturbances have a five-fold increased risk of developing another UL in the short- to mid-term, compared to those with adequate nutrition [13]. A 24-hour urine and metabolic study is useful to evaluate the presence of metabolic abnormalities, which could represent a risk for recurrence, therefore enabling its prevention [13]. It is recommended that all children with a history of UL should remain under medical care to repeat metabolic tests at least once a year, as they could show different patterns with time [12].

Due to the recurrence of the disease, it is essential to introduce a prophylactic treatment after the removal of kidney stones [14]. The main recommendation for prophylactic treatment is adequate fluid intake, which should be consumed evenly throughout the day, mainly in the evening. As for dietary recommendations, calcium intake should not be restricted since calcium ions partially bind to oxalates in the gastrointestinal tract, thus preventing secondary hyperoxaluria. Saline intake should be limited so as not to exceed a urinary sodium excretion of 130 mmol/l. Animal protein intake should also be limited, as excessive intake increases calciuria, reduces citrate excretion, and favors the crystallization of calcium oxalate on sodium urate crystals. Finally, sweets and dietary supplements should be avoided. For conservative treatment, if the metabolic examination is accompanied by hypocitraturia or hypomagnesuria, the administration of potassium or magnesium citrate with vitamin B6 is recommended. Similarly, if UL is very symptomatic or if it is accompanied by a decrease in bone mineral density, the administration of hydrochlorothiazide is recommended to reduce calcium excretion [12].

As for invasive treatment, mini-ECIRS has proven to be a novel technique of renal puncture and safe fragmentation, which increases surgical success while maintaining a high stone-free rate, reduces postoperative pain, and avoids postoperative complications such as renal bleeding, ureteral injury, and renal dysfunction, so it could be considered the best therapeutic option to treat large renal stones in pediatric patients [15,16].

There is a pressing need to direct research efforts toward comprehending the risk factors for calcite stone formation and recurrence, as well as its impact on the risk of acute kidney injury and chronic kidney disease in the pediatric population.

## Conclusions

Calcium carbonate calculi are exceedingly rare, even more so in pediatric patients. Stone composition and metabolic studies should be conducted routinely to identify abnormalities and prevent lithiasis recurrence. Currently, there are no studies about the correlation between risk factors and calcite stone formation and recurrence, making this a promising area for pediatric and urological research.
